# Effect of Lubricant Type on the Friction Behaviours and Surface Topography in Metal Forming of Ti-6Al-4V Titanium Alloy Sheets

**DOI:** 10.3390/ma14133721

**Published:** 2021-07-02

**Authors:** Marcin Szpunar, Tomasz Trzepieciński, Krzysztof Żaba, Robert Ostrowski, Marek Zwolak

**Affiliations:** 1 Doctoral School of Engineering and Technical Science, Rzeszow University of Technology, al. Powst. Warszawy 12, 35-959 Rzeszów, Poland; d547@stud.prz.edu.pl; 2 Department of Materials Forming and Processing, Rzeszow University of Technology, al. Powst. Warszawy 8, 35-959 Rzeszów, Poland; rostrows@prz.edu.pl (R.O.); m.zwolak@prz.edu.pl (M.Z.); 3 Department of Metal Working and Physical Metallurgy of Non-Ferrous Metals, Faculty of Non-Ferrous Metals, AGH—University of Science and Technology, al. Adama Mickiewicza 30, 30-059 Cracow, Poland; krzyzaba@agh.edu.pl

**Keywords:** friction, friction mechanisms, sheet metal forming, titanium sheets

## Abstract

The aim of the research described in this paper is to analyse the synergistic effect of types of synthetic oil and their density on the value of the coefficient of friction (COF) of Ti-6Al-4V titanium alloy sheets. Lubrication performance of commercial synthetic oils (machine, gear, engine and hydraulic) was tested in a strip draw friction test. The friction tests consisted of pulling a strip specimen between two cylindrical fixed countersamples. The countersamples were placed in the simulator base mounted on a uniaxial tensile test machine. Due to the complex synergistic effect of different strip drawing test parameters on the COF, artificial neural networks were used to find this relationship. In the case of both dry and lubricated conditions, a clear trend was found of a reduction of the coefficient of friction with nominal pressure. Engine oil 10W-40 was found to be the least favourable lubricant in reducing the coefficient of friction of Grade 5 titanium sheets. The two main tribological mechanisms, i.e., galling and ploughing, played the most important role in the friction process on the test sheets. In the range of nominal pressures considered, and with the synthetic oils tested, the most favourable lubrication conditions can be obtained by using a type of oil with a low viscosity index and a high kinematic viscosity.

## 1. Introduction

The production of lightweight structures in the automotive and aviation industries requires the use of materials with high strength in relation to the density of the material used. In addition, aeronautical engineering requires corrosion resistance at elevated temperatures [1]. Such requirements are met by commonly manufactured titanium alloys. The shaping of titanium alloys takes place in plastic working processes such as rolling, forging, drawing, extrusion, deep drawing or spinning. Titanium alloy sheets are defined as hard-to-form materials with respect to their high strength and formability characteristics [2]. Moreover, the hexagonal-close packed crystal structure of titanium alloys, in which only the basal plane can move, exhibits a low ductility and formability at room temperatures [3]. The limited wear resistance of titanium alloys makes them difficult to form because they are notoriously susceptible to galling in contact with other surfaces [4,5]. Therefore, titanium alloy sheets require other forming parameters than the commonly formed steel sheets.

Friction is a phenomenon inseparable from plastic forming processes [6]. It is one of the most important factors that affects tribological processes and stress distribution and has a significant impact on the roughness of the formed surface thus obtained [1,7]. In order to be able to predict the value of coefficient of friction (COF) in analyses of sheet-forming processes, it is necessary to carry out the tribological test for a given pair of mating elements (tool and plate) [8]. The COF is mainly influenced by the roughness of the tool and of the material formed, lubrication conditions [9], mechanical properties of the sheet metal [10], character of contact (static/dynamic) [11] and temperature [12].

The primary way to reduce friction is to use lubricants. Among the range of industrial oils and greases with a wide range of parameters, the selection of the appropriate lubricant for a given application becomes problematic. Titanium is known to exhibit high friction with conventional lubricants, and is prone to seizing and galling. Any lubricant proposed to improve the lubrication of titanium and its alloys must not seriously impair either the corrosion resistance or the surface roughness of the workpiece. Titanium and its alloys are materials with low drawability and low wear resistance. Both groups of materials show a high tendency to induce sticking on the working surfaces of tools, which sometimes simply makes the forming process of the sheets difficult, and sometimes even prevents it taking place [13].

Currently, the tribology of titanium sheets is the subject of many investigations in the context of forming temperature [14], lubricant type and contact pressures [15]. Adamus et al. [16] investigated the effect of anti-adhesive coatings on steel tools in the sheet forming of titanium plates. The research showed a significant improvement in the surface roughness parameters of the formed surface with the use of multi-layer physical vapour-deposited coatings on the tool, and thus possible complete elimination of lubricants in the future. Więckowski and Adamus [17] studied the tribological properties and COF of titanium alloy sheets on a roller-block tester, focusing research on the selection of appropriate lubrication, surface treatment of materials and the use of anti-adhesive coatings. The test results obtained indicate the unfavourable tribological properties of the test materials, thus demonstrating the necessity to conduct further tests in order to increase the wear resistance and to reduce the COF. Jozwik [18] also evaluated the tribological properties of Ti-6Al-4V titanium alloy in the pin on disk test.

The authors of the studies in the literature on the friction of titanium and its alloys focus almost exclusively on testing various lubricants in order to minimise frictional resistance without looking for complex interactions between the properties of oils and the value of the COF. In this paper the synergistic effect of types of synthetic oil and their density on the value of the COF of Ti-6Al-4V titanium alloy sheets was analysed. The lubrication performance of commercial synthetic oils (machine, gear, engine and hydraulic) was tested in a strip draw friction test. The results of the strip draw test (SDT) were processed by artificial neural networks (ANNs) to find the interactional effect of lubricant density and their kinematic viscosity on the value of the COF. A study was also carried out on friction mechanisms, relating them to lubricant type and load.

## 2. Materials and Methods

### 2.1. Material

Ti-6Al-4V (Grade 5) titanium sheets with a thickness of 0.5 mm supplied in annealed thermal treatment state, were used as a test material. The test materials were delivered by Timet (Warrensville Heights, OH, USA). Grade 5 is pure alpha-beta titanium with aluminium as the alpha stabiliser and vanadium as the beta stabiliser.

**Figure 1:**
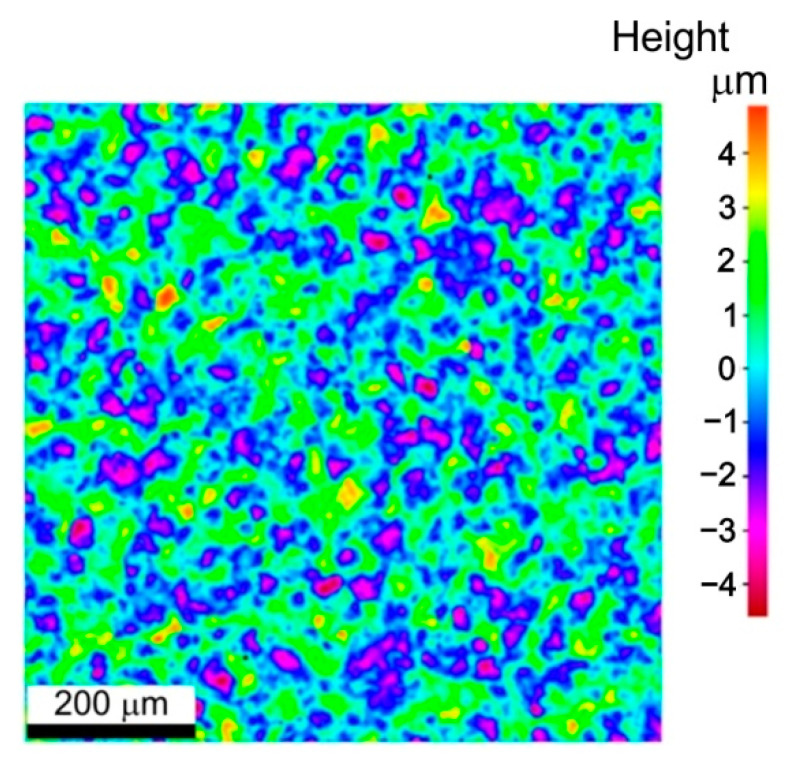
Topography of the as-received Grade 5 titanium alloy surface.

The surface roughness parameters were measured using an Alicona InfiniteFocus G4 non-contact optical 3D surface characterisation and measuring tool (Alicona Imaging GmbH, Raaba/Graz, Austria). Topographies of the specimens were characterised by the well-known 3D roughness parameters: Sa, Sp, Sv, Sz, Ssk and Sku. The as-received surfaces and sheet surfaces after the friction test were subjected to roughness measurements. The measurement principles are ISO-certified in EN ISO 25178 [19]. The surface roughness parameters of the as-received surface (Figure 1) are as follows: Sa = 1.13 μm, Sz = 9.48 μm, Sp = 4.88 μm, Sv = 4.60 μm, Ssk = 0.16 μm and Sku = 2.91 μm.

**Figure 2:**
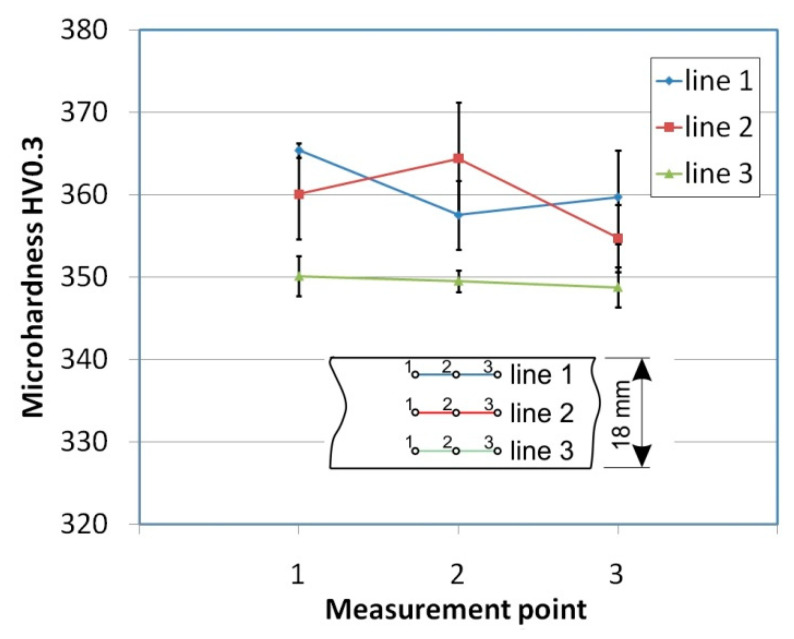
Results of the microhardness measurements.

To determine the microhardness of the sheet material, a NEXUS 4303 tester (INNOVATEST Europe BV, Maastricht, The Netherlands) with a Vickers pyramidal diamond tip was used. The microhardness HV0.3 was measured in nine locations on the sheet surface, evenly distributed over the width and length of the sample (Figure 2). To limit the influence of roughness on the microhardness value, the sheet surfaces were polished. Average values of the three measurements of microhardness along each line (Figure 2) showed a random character with no clear directional properties.

The as-received surfaces and sheet surfaces after the friction tests were examined in detail using a Hitachi model S-3400N (Hitachi, Chiyoda, Japan) variable pressure scanning electron microscope (SEM) equipped with a Back-Scattered Electron (BSE) detector (Hitachi, Chiyoda, Japan). BSE imaging was conducted with a 20 kV accelerating voltage.

### 2.2. Experimental Testing

**Figure 3:**
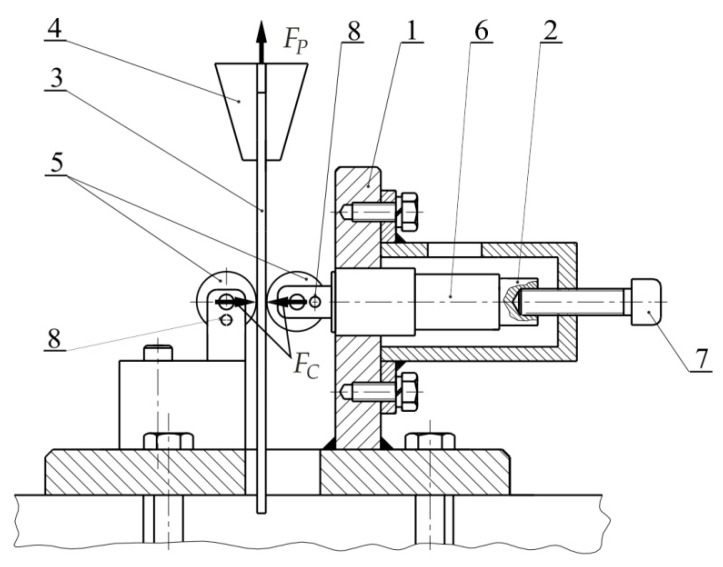
Principles of strip drawing test 1—base, 2—teflon insert, 3—specimen, 4—upper grip of testing machine, 5—cylindrical rolls, 6—mandrel, 7—set screw, 8—fixing pin, *F_p_*—friction force, *F_c_*—clamping force.

The COF of the test sheets were determined using the Strip Drawing Test SDT. A schematic diagram of the SDT-based friction simulator is shown in Figure 3. The simulator was mounted in the lower grip of the Zwick/Roell uniaxial tensile test machine (Zwick/Roell, Ulm, Germany). The SDT consisted in pulling an 18-mm-wide strip specimen between two fixed countersamples. After placing the sample between the counter-samples, its upper end was fixed in the upper grip of the testing machine. In this way, the value of the friction force was recorded by the measuring system of the testing machine. The clamping force was applied through the set screw with a torque wrench in such a way as to obtain different levels of clamping force *F_C_*.

Tests were conducted at room temperature. The COF *μ* has been evaluated based on the ratio of the clamping (horizontal) force *F_c_* and the friction (vertical) force *F_p_*:

(1) $$\mu =\frac{F_{p}}{2F_{c}}$$

**Figure 4:**
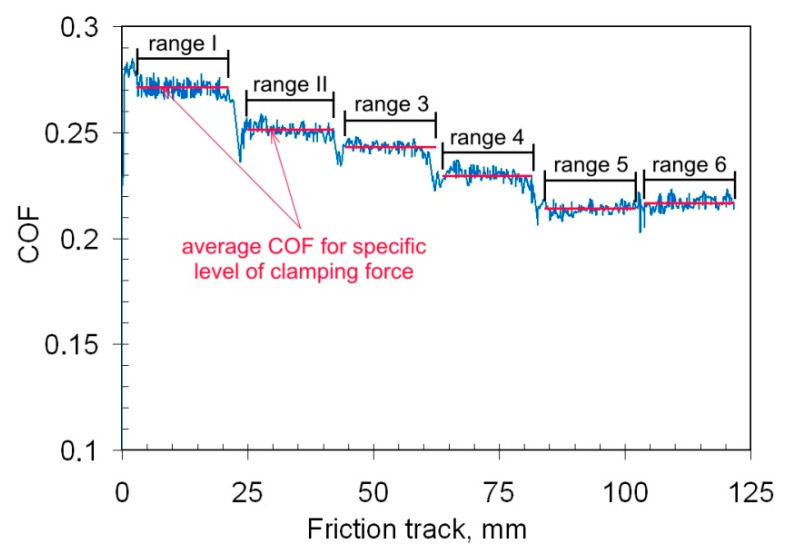
Variation of the coefficient of friction during the friction test with lubrication using L-AN 46 machine oil.

The clamping force value was increased sequentially during the tests. One sample permits the determination of the values of the COF for six levels of clamping force. The average value of the COF was determined separately for all levels of variation of the COF (ranges 1–6 in Figure 4). At all levels of clamping force, the strip specimen was drawn for a distance of about 0.02 m, and about *i* = 350–400 discrete values of the COF have been obtained. The average COF *μ_av_* for a specific level of clamping force has been determined using the formula:

(2) $$\mu_{av}=\frac{1}{i}\underset{i}{\sum }\mu_{i}$$

where *i* is the value of COF determined for the specific level of constant clamping force.

In the following parts of this manuscript, the average value of COF *μ_av_* will be considered.

The surface roughness of the countersamples was Sa = 0.43 μm. The friction tests were carried out in both dry and lubricated conditions. Before the tests, the surface of the as-received surfaces was cleaned using acethone. In lubricated conditions, petroleum oils were used: machine oil L-AN 46 (Orlen Oil, Kraków, Poland), gear oil 75W-85 (EXXON Mobil Corp., Irving, TX, USA), engine oil 10W-40 (BP Europe SE, London, UK) and hydraulic oil Hydrol L-HL 46 (Orlen Oil, Kraków, Poland) The engine and gear oils used in the tests are marked with a numerical code system of the Society of Automotive Engineers (SAE) for grading motor oils according to their viscosity characteristics. SAE distinguishes 6 “winter” (W) viscosity classes marked with a number before the letter W (0W, 5W, 10W, 15W, 20W, 25W) and 8 “summer” classes with a number after the letter W (W8, W12, W16, W20, W30, W40, W50, W60). The document SAE J300 [20] defines the viscometrics related to these grades. The higher the number, the more viscous the oil is. Gear oils are classified in a similar way in accordance with the SAE J306 [21] standard, which distinguishes 9 classes ranked in a scale of increasing viscosity: 70, 75, 80, 85, 90, 110, 140, 190, 250. The designations of universal oils are two numbers separated by the letter W. The number before W indicates the winter viscosity class and the number after the W indicates the summer viscosity class. According to the data of the manufacturers of oils, classes 10W-40 and 75W-85 correspond to the kinematic viscosities of 105.3 and 64.6 mm^2^/s, respectively. The basic properties of synthetic oils used are listed in Table 1.

Due to their availability, synthetic oils are commonly used in sheet metal forming. In order to reduce the number of experiments, one oil from the group of different oils (hydraulic, gear, etc.) was selected. The strip specimens were lubricated using a teflon shaft (Poliamid 24, Bydgoszcz, Poland) [11]. The amount of lubricant applied to each of the two surfaces of the samples was 2 g/m^2^ [11].

Contact pressure in the contact of the cylindrical roller and flat specimen has been evaluated according to the formula [22]:

(3) $$p=\sqrt{\frac{0.418^{2}\times F_{c}\times E}{w\times R}}$$

where *E* is Young’s modulus of sheet material (115 GPa), *w* is the specimen width, *R* is the radius the roller.

### 2.3. Artificial Neural Network Modelling

Due to the complex relationships between the friction parameters and the value of the COF, ANNs were used to find the nature of these relations. A multilayer neural network was used to determine the relationship between the friction process parameters and the value of the COF. The analyses were performed with the use of the Statistica program (StatSoft Inc., release 4.0 E, 1998, Tulsa, OK, USA). The input parameters include the oil density, kinematic viscosity, viscosity index and nominal pressure. The output value was the value of the COF. The values of the input parameters and the COF have been normalised to the range [−1, +1] suggested in the literature [23,24,25]. Using the Intelligent Problem Solver built into Statistica, many experiments were carried out with many network structures and different values of neurons in the hidden layer. Networks with one hidden layer were only analysed because a network with this structure is capable of investigating any complex problem [26].

The back propagation algorithm was used to train the network. The input dataset containing all the COF results obtained under lubricated conditions was divided into two sets. The training set contained 90% of the data and the validation set 10% [27]. The network quality was assessed on the basis of the value of the coefficient of determination R^2^ for the training set and the standard deviation ratio SDR [27]:

(4) $$\mathrm{SDR}=\frac{\mathrm{SDE}}{\mathrm{SDC}}$$

where SDE—standard deviation of errors, SDC—standard deviation of value of COF.

## 3. Results and Discussion

### 3.1. Coefficient of Friction

**Figure 5:**
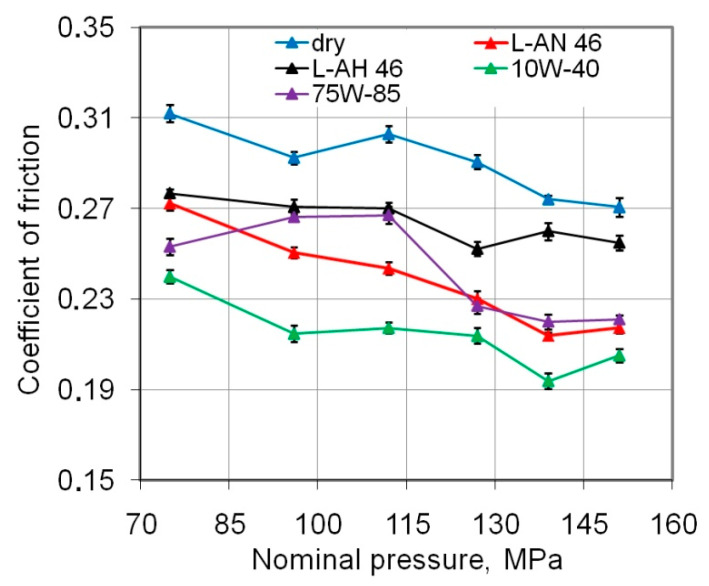
Effect of friction conditions on the value of the COF.

In the investigations, nominal pressures in the range 75 to 151 MPa (Figure 5) were considered, which is fully consistent with the range of pressures existing in sheet metal forming as noted in the wide literature review prepared by Cillauren et al. in [28].

Due to the non-linear relationship between the clamping force and friction force, the value of COF decreases with increasing pressure. This relationship, typical for a strip-drawing test with cylindrical counter-samples, was also found by Kirkhorn et al. [29] Reduction of COF with an increasing load is found for both dry and lubricated conditions. This may be because the contact area grows disproportionately to the increase in load. In sheet metal forming processes, in which the relatively soft sheet is deformed by a hard tool, the contact area is crucial in assessing the value of overall frictional resistance of the tribological system [30].

The greatest reduction in COF in the whole range of nominal pressures considered was observed during friction with 10W-40 engine oil (Figure 5). The change in COF for the friction process produced using 75W-85 gear oil shows a quite different character from other lubricants. This oil is characterised by a lower viscosity (64.6 mm^2^/s) than 10W-40 engine oil (105.3 mm^2^/s) and a higher density than L-HL 46 oil (44.2 mm^2^/s). An oil with an unfavourable viscosity value may not have formed a lubricant pad that could separate the peaks of the roughness at low pressure values. After exceeding a certain pressure value of 114 MPa, it resulted in a flattening of the roughness asperities and the creation of a lubricant pressure, effectively reducing the degree of contact between the contacting surfaces. Therefore the COF for higher pressures have a much lower value.

### 3.2. Effectiveness of Lubrication

To evaluate the lubrication efficiency, a coefficient value relating to the effectiveness of lubrication *ε_l_* (Equation (5)) is introduced [11]:

(5) $$\varepsilon_{l}=\frac{\mu_{dry}-\mu_{oil}}{\mu_{dry}}\times 100\%$$

where *μ_dry_* and *μ_oil_* are the COFs determined in dry and lubricated conditions, respectively.

**Figure 6:**
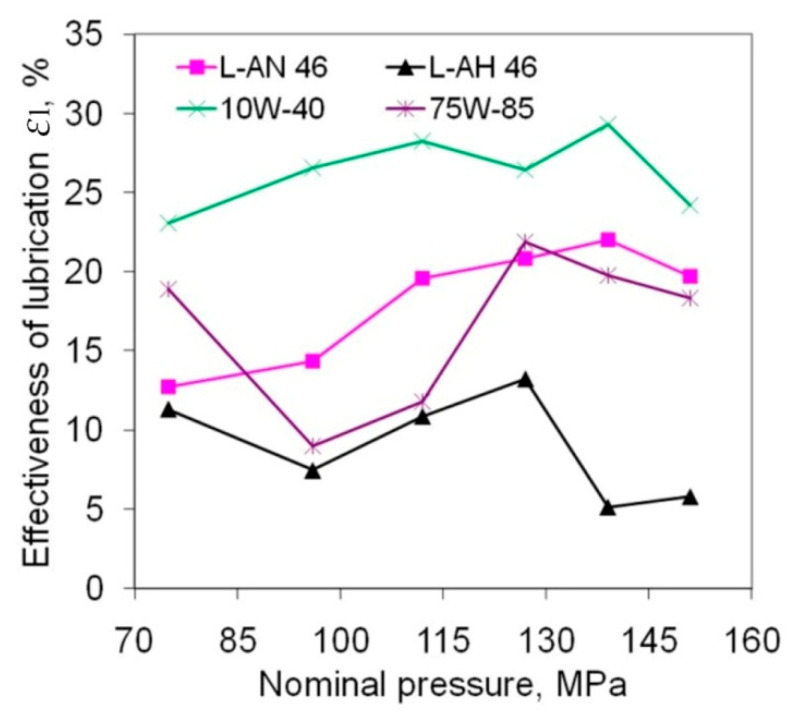
Effect of friction conditions on the effectiveness of lubrication.

The test lubricants used to reduce the frictional resistance of the titanium alloy sheet showed a reduction in the COF in the range between about 4.5 and 29% (Figure 6). Engine oil 10W-40 showed the best lubricating properties. The lubrication efficiency of this oil is between 23 and 29%. The effectiveness of lubrication for engine oil 10W-40 is quite stable across the whole range of nominal pressures studied.

A small tendency to increase the ε*_l_*-value with an increase in nominal pressure is observed and L-AN 46 machine oil shows a similar trend while 75W-85 gear oil showed the greatest sensitivity of lubrication efficiency to pressure value. Great variation is found in the effectiveness of lubrication across the whole range of nominal pressures utilised.

Hydraulic oil L-HL 46 showed distinctly poor properties in reducing the COF of Ti-6Al-4V sheet in the range of pressures used in the test. This oil is characterised by a similar density and viscosity to L-AN 46 oil. However, the viscosity index of L-HL 46 oil is 101 while the index for L-AN 46 oil is 94. This therefore suggests that the viscosity index has an important role in reducing friction during Sheet Metal Forming (SMF). In order to effectively reduce the COF as a result of lubrication, it is necessary to generate appropriate pressure in the lubricant layer [31,32].

### 3.3. Surface Roughness

**Figure 7:**
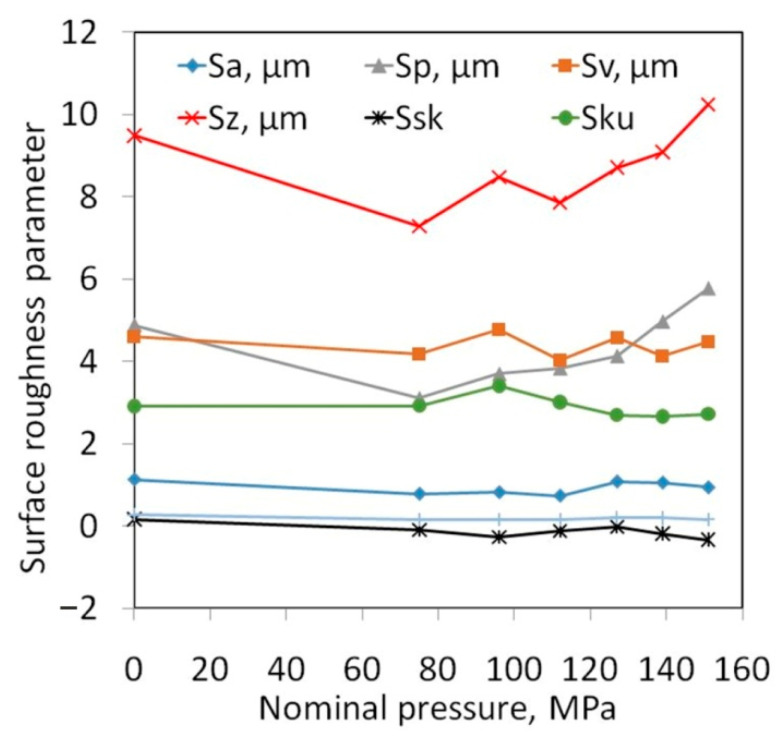
Influence of nominal roller pressure on the value of the Sa, Sp, Sv, Sz, Ssk and Sku parameters.

Dry friction conditions are preferable for sheet metal forming due to the economy of the production process. In contrast, a lack of lubricant causes the most heavy-duty conditions to form. Therefore, for analysis of the change in surface topography of Ti-6Al-4V, sheets tested in dry friction conditions were selected. The increase in the nominal pressure value leads to a reduction in the values of Sku and Ssk. In the range of normal pressures up to 139 MPa, the friction process led to a decrease in the Sz parameter. In general, the values of surface roughness parameters Sp, Sz, Sa and Sv increased with the value of nominal pressure (Figure 7).

**Figure 8:**
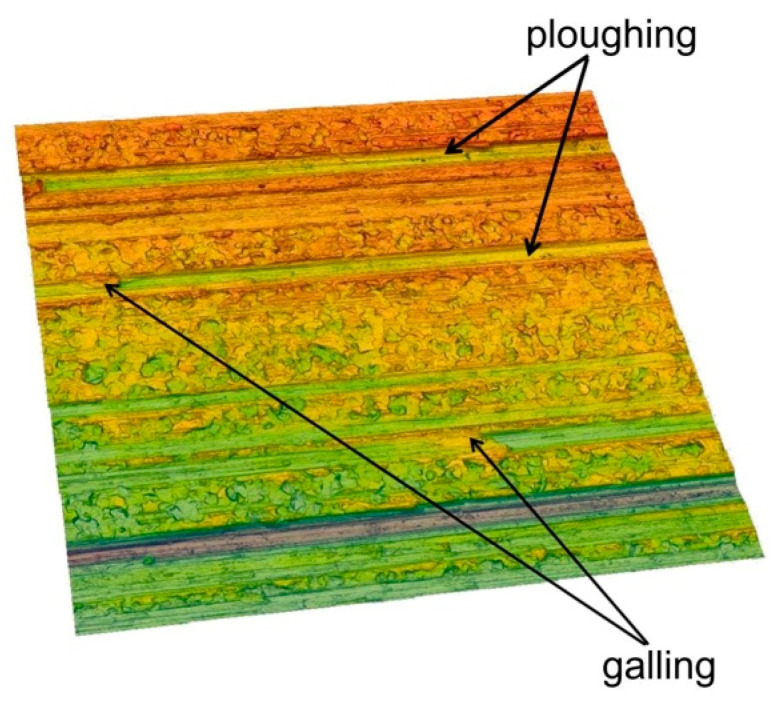
The surface topography of the sheet after a strip drawing test in dry friction conditions, nominal pressure 151 MPa.

However, for the highest pressure analysed of 151 MPa, the interaction of the surface roughness of the countersample causes ploughing of the specimen surface by products of galling (Figure 8) and the roughness asperities of the cylindrical countersample. Therefore, the 10-point peak-valley surface roughness Sz increases more than the value of the Sz parameter for the as-received surface. Galling and creation of protrusions on the tool surface are the main problems arising during working Ti-6Al-4V titanium alloy. Poor abrasion resistance of titanium alloys has been observed by Budinski [33]. Poor resistance to abrasive wear of sheet surface is attributed to its inability to maintain an oxide film on the surface, which then causes direct metallic contact. This leads to higher friction, flattening and ploughing of the workpiece surface. A passive thin oxide film protects titanium alloys against corrosion. In addition to TiO_2_, the passive layer contains oxides of the other elemental constituents of the alloy [34].

Abrasive wear dominates when forming relatively soft materials using tools with a high surface roughness. If the sheet surface is loaded with stresses exceeding the yield point of the sheet material, then the surface roughness asperities flatten. The sheet surface roughness distortion causes a poor quality of the final product, while the presence of closed lubricant pockets leads to an incorrect lubrication.

**Figure 9:**
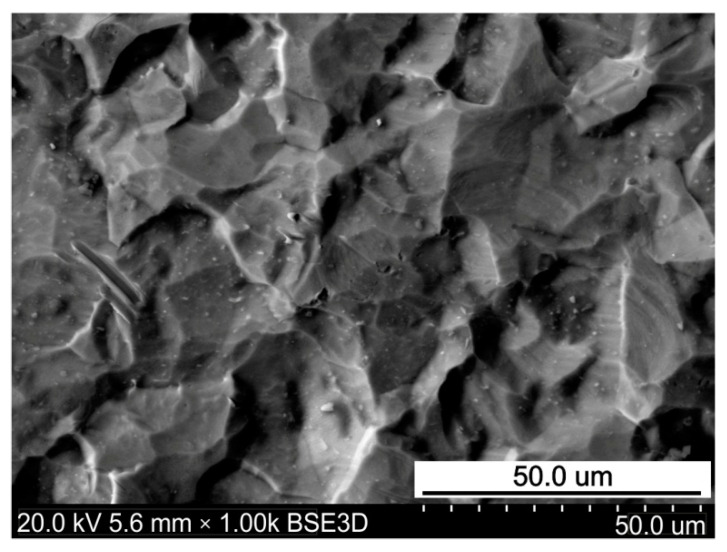
SEM micrograph of the as-received surface.

**Figure 10:**
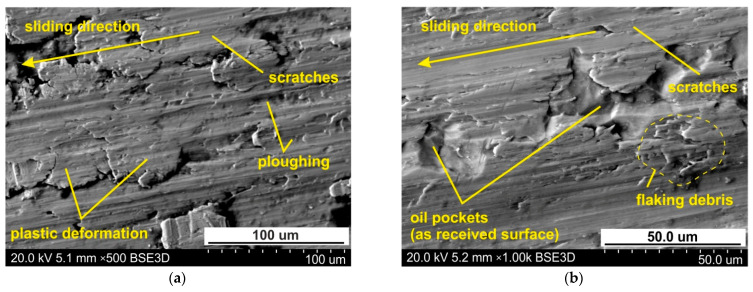
The specimen surfaces tested at 96 MPa in (**a**) dry friction and (**b**) lubricated conditions using engine oil 10W-40.

The Ti-6Al-4V titanium alloy sheet is susceptible to surface flattening. The as-received surface (Figure 9) exhibits a regular grain structure with quite a smooth surface of grains. However, after both lubricated and dry conditions, intensive flattening of the sheet surface was observed. Friction forces acting between the surfaces of the tool and workpiece caused ploughing of the tool surface asperities in the surface of the sheet (Figure 10a). The second mechanism, which is problematic when forming titanium sheets, is galling, which is due to lubricant film breakdown, leading to scoring and bad surface quality [35].

**Figure 11:**
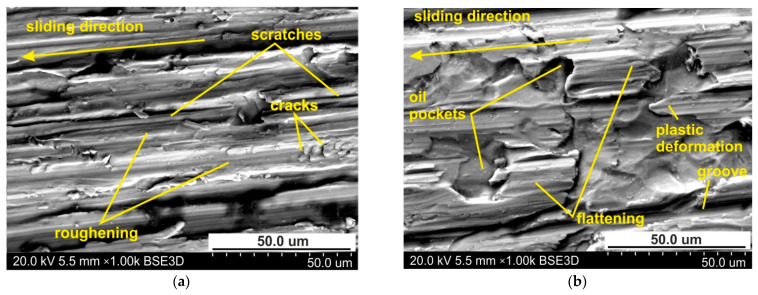
The specimen surfaces tested at 114 MPa in (**a**) dry friction and (**b**) lubricated conditions using machine oil L-AN 46.

**Figure 12:**
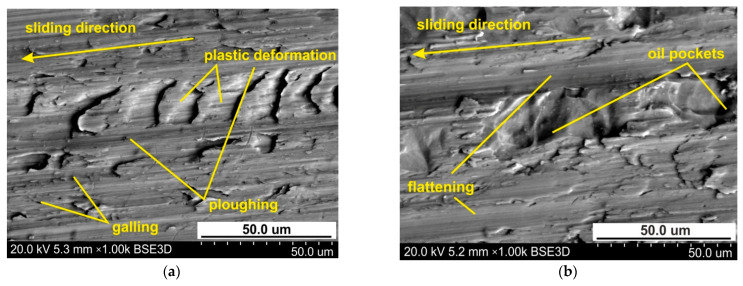
The specimen surfaces tested at 127 MPa in (**a**) dry friction and (**b**) lubricated conditions using engine oil SAE 10W-40.

When lubricating the sheet surface with 10W-40 engine oil, the ploughing mechanism is also active, but to a lesser extent than under lubrication conditions. In this way, oil pockets in the valley surface corresponding to the as-received surface are visible on the surface (Figure 10b). This conclusion can be applied to all the lubrication conditions. Figure 11 shows the specimen surfaces tested at 114 MPa using engine oil SAE 10W-40. The greater the pressure, the more active are the roughening and galling mechanisms which cause local adhesion of material layers to the tool. In this way, the material of the top layer moves with the tool, causing cracks and layering of the flattening of the material (Figure 11a,b). Increasing the load intensifies the plastic deformation of sheet surface in dry friction conditions (Figure 12a,b).

**Figure 13:**
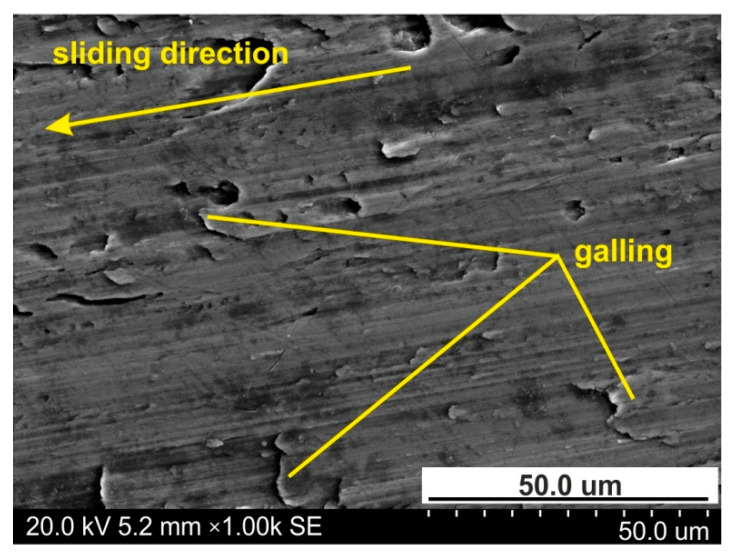
The specimen surfaces tested at 139 MPa in dry friction.

Galling phenomena between the workpiece and tool lead to deterioration on the surface of finished products (Figure 13) and severe wear of the tools. The galling phenomenon is made clearer by measuring the heat generated by friction and contact pressure [36]. Galling is a consequence of adhesive wear, which is defined as the separation of material particles due to adhesive tacking on the contact surfaces. Initially, this type of wear is localised only within single asperities and occurs with a constant intensity. With increasing pressure, galling regions are formed, which begin to propagate to the subsurface layer [37].

**Figure 14:**
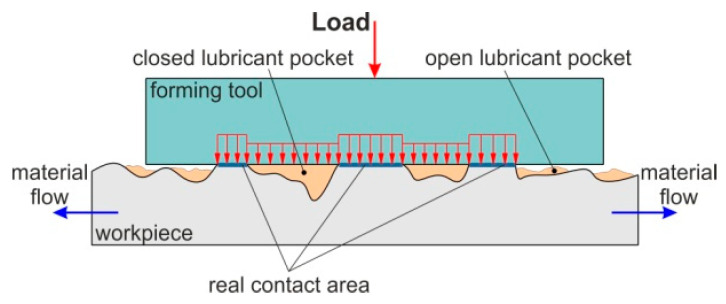
Closed and open lubricant pockets.

During loading, the asperities deform elastically or plastically, effecting the surface topography of sheet metal and the real area of contact. For elastic-plastic metals, junction growth during an imposed sliding motion results in an increased real contact area, which may lead to a decrease in the volume of lubricant pockets [38]. Under load, the pressure of lubricant increases, and the lubricant is trapped in the roughness valleys. The open oil pockets (Figure 14) located at the edges of the surface are not capable of holding lubricant during the friction process. Closed oil pockets are separated from the outer edges of the material and store the lubricant in the closed volume of the valleys. The lubricant closed in valleys is a kind of hydrostatic cushion that takes a part of the load [38]. According to the lubrication pocket theory developed by Vollertsen [39], there is an increase in COF in open lubrication theory whereas there is a decrease in COF in the case of closed lubrication theory.

The influence of nominal pressure and friction conditions on the occurrence of mechanisms accompanying the friction process is presented in Table 2. Determining the intensity of a given mechanism and the boundaries of occurrence of individual mechanisms may be subject to noise due to subjective observation errors. In lubricated conditions, flattening of surface asperities is a dominant mechanism of friction in terms of nominal pressures between 75 and 127 MPa (Table 2). Increasing nominal pressure has led to the intensification of ploughing. Under dry friction, the ploughing mechanism has already been observed from a pressure of 96 MPa. Galling is clearly observed in dry friction conditions with pressures of 139 and 151 MPa.

During sheet metal forming, the adhered work hardened workpiece material represents a hard abrasive particle, which can cause scratches on the sheet surface [11,40]. Adhered hard material deteriorates the surface topography of the tool. In consequence, the disturbed surface of the tool may indent or scratch the workpiece surface and thereby worsen the quality of the drawpiece in SMF [41]. The phenomenon that limits the formation of titanium sheets used in the aerospace industry by sheet forming is the adhesion of titanium particles to the tool surface, which intensifies the decline in the quality of the surface of the sheet metal. Despite the fact that the sheets were subjected to high nominal pressures, oil pockets are visible on their surfaces (Figure 10b). As pockets bring lubricants into the friction region, they may reduce friction under these conditions.

Wear of sheet surface could occur due to both abrasive and adhesive mechanisms. In SMF, abrasive wear occurs when a hard surface of tool cuts material away from a softer workpiece. Adhesive wear is a type of wear due to localized bonding between contacting solid surfaces, leading to material transfer between two surfaces or loss from either surface [42]. Hence, titanium alloys are particularly prone to adhesive wear [43,44], leading to galling (Figure 13).

The friction and wear effects are observed due to the adhesion effects between asperities, the ploughing at contacting asperities, and the hydrodynamic friction stresses that appear when the lubrication regime is applied [12]. The wear mechanisms identified in the strip drawing test were distinguished into a sequence of events consisting of initial local adhesive wear of the sheets resulting in transfer of sheet material to the tool surfaces. Successive increasing of the nominal pressure led to a growth of the transfer layer and initiation of scratching of the sheet metal (Figure 10). This observation is in line with the results found by Gåård [38] who indicated that scratching changed into severe adhesive wear, associated with gross macroscopic damage.

For the forming industry, wear and surface damages such as ploughing and adhesion of sheet material is detrimental for the tool performance. In the interaction between the tool and the sheet, the tool surface is stationary, while the sheet surface is renewed at every new forming operation. The sheet metals possess a relatively rough surface and wear in sheet metal forming is stochastic in nature and tool life length predictions are difficult to make [38].

### 3.4. Artificial Neural Networks

First, an attempt was made to take into account four parameters in the network input, i.e., oil density, kinematic viscosity of the oil, viscosity index and nominal pressure. The analyses carried out in the Statistica program showed that the network taking into account all four parameters at the input does not provide an acceptable value of the determination coefficient for the training set. Depending on the number of neurons in the hidden layer in the range 5–15, the value of the R^2^ coefficient was in the range between 0.564 and 0.735. The explanation for this may be that there is too small a number of training data in relation to the number of input parameters or data noise due to the correlation between input parameters. In subsequent studies, it was decided to limit the number of input variables to three, in accordance with Table 3.

The network architecture was selected independently for all ANN1-ANN4 network models (Table 3). Table 4 shows the architectures (number of input neurons—number of neurons in the hidden layer—number of neurons in the output layer) of networks that ensure the highest value of the coefficient of determination for the training set and the corresponding values of basic regression parameters.

The R^2^ value was lowest, with a network with no nominal pressure at the input. This proves that nominal pressure has a dominant role in the value of the COF. The low quality of this network is also demonstrated by the value of the SDR. This parameter takes values in the range [0, 1]. The lower the SDR value, the better the prognostic quality of the ANN model [11]. Out of all the input parameters analysed, a set of three parameters, i.e., kinematic viscosity, viscosity index and nominal pressure, gave the best regression parameters in the form of the highest R^2^-value and the lowest SDR value. These parameters correspond to the ANN1 network (Table 4).

**Figure 15:**
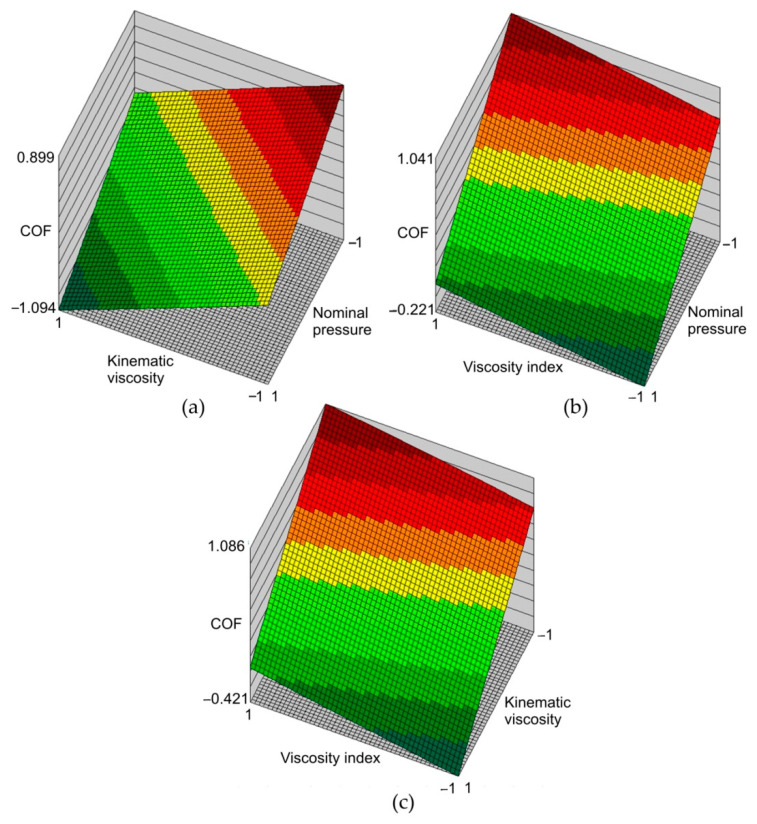
Response surfaces (ANN1) of the effect of (**a**) kinematic viscosity and nominal pressure, (**b**) viscosity index and nominal pressure, and (**c**) kinematic viscosity and viscosity index on the value of COF (normalised data).

Figure 15 shows the response surfaces for the network ANN1 in the form of relationships between the input parameters and the COF value. The range of changes of input parameters in the response surfaces correspond with the range of changes of parameters used during the experimental tests. Therefore, the interpretations of the response surfaces are valid only for the limited range of changes of input parameters. It is clear from Figure 14a and Figure 15b that with an increase in nominal pressure, the value of the COF decreases. This is in line with the trend of experimental results in Figure 5. Increasing the viscosity index with a simultaneous decrease in nominal pressure (Figure 15b) and kinematic viscosity (Figure 15c) increases the value of the COF. The most favourable lubrication conditions with the synthetic oils tested can be obtained by using a type of oil with a low viscosity index and a high kinematic viscosity (Figure 15c). In relation to the COF, the high viscosity of the lubricant is most effective in reducing the COF (Figure 15a). For each value of the pressures analysed, the increase in viscosity index leads in an increase in COF (Figure 15b). An inverse relationship occurs between kinematic viscosity and COF (Figure 15a).

## 4. Conclusions

In this manuscript, the frictional phenomena of Ti-6Al-4V titanium alloy sheets were tested in an SDT using a tribological simulator. The following conclusions, valid for the test conditions used in this study, are drawn from the research:

- A general trend for a slight reduction in the value of the COF with increasing load was found. This observation applies to all lubrication conditions and dry friction conditions.
- Synthetic engine oil 10W-40 was found to be the most favourable lubricant for reducing the COF of Ti-6Al-4V titanium alloy sheets.
- When high pressures are applied, ploughing and galling mechanisms were observed which are typical when forming titanium sheets.
- Significant decreases have been observed in the values of the Sz and the Sp parameters in the range of nominal pressure between 75 and 114 MPa.
- The 10W-40 engine oil was the most stable in providing effective lubrication over the whole range of pressures applied.
- In order to minimise the COF of Ti-6Al-4V titanium alloy sheets, oil with high kinematic viscosity and a low viscosity index should be used.
- Decreasing the kinematic viscosity of the oil increases the COF of Ti-6Al-4V sheets.

The high values of COF resulting from the contact of the Ti-6Al-4V sheet metal with the steel counter-sample in the range of nominal pressures considered confirms the high susceptibility of the titanium alloy to deterioration in the surface quality of the sheet as a result of flattening, ploughing and galling. Although 10W-40 engine oil proved to be the most beneficial in reducing the COF, adhesive wear from the sheet surface was observed with all lubricants from the smallest pressures considered. Thus, if the basic quality criterion of the product is surface quality, the lubricants used determined the nature of the changes in the sheet surface topography to a similar extent. Nevertheless, reducing the value of the COF may favourably affect increases in the limit strains during sheet forming.

## Figures and Tables

**Table 1:** Basic properties of oils used.

Oil Type	Viscosity Index	Kinematic Viscosity, mm^2^/s	Density, kg/m^3^
Engine oil SAE 10W-40	156	105.3	872
Hydraulic oil L-HL 46	101	44.2	877
Gear oil SAE 75W-85	169	64.6	837
Machine oil L-AN 46	94	43.9	875

**Table 2:** The effect of friction conditions on the dominant tribological mechanism (flattening—F, galling—G, ploughing—P).

Friction Conditions	Nominal Pressure, MPa					
	75	96	114	127	139	151
dry friction	F	P	P	P	G	G
L-AN 46	F	F	F	P	P	P
L-HL 46	F	F	F	P	P	P
75W-85	F	F	F	P	P	P
10W-40	F	F	F	P	P	P

**Table 3:** Input parameter in artificial neural networks.

ANN Model	Input Parameters			
	Density of Oil	Kinematic Viscosity	Viscosity Index	Nominal Pressure
ANN1	no	yes	yes	yes
ANN2	yes	no	yes	yes
ANN3	yes	yes	no	yes
ANN4	yes	yes	yes	no

**Table 4:** Basic regression statistics of the ANNs analysed.

Parameter	ANN Structure			
	ANN1 3-8-1	ANN2 3-9-1	ANN3 3-11-1	ANN4 3-8-1
Error mean	0.07576	0.268	−0.239	−0.429
Standard deviation of error	0.209	0.343	0.321	0.428
Absolute error mean	0.178	0.381	0.306	0.481
SDR	0.331	0.603	0.528	0.745
R^2^	0.945	0.801	0.849	0.667

## Data Availability

The data presented in this study are available on request from the corresponding author.

## References

[1] Shen H., Wang L. (2021). Corrosion resistance and electrical conductivity of plasma nitrided titanium. Int. J. Hydrogen Energy.

[2] Djavanroodi F., Janbakhsh M., Sieniawski J., Ziaja W. (2013). Formability Characterization of Titanium Alloy Sheets. Titanium Alloys-Advances in Properties Control.

[3] Park N.K., Park J.G., Seo S.H., Kim J.H. (2010). Drawability of Ti-6Al-4V Sheet at Elevated Temperatures. Mater. Sci. Forum.

[4] Wiklund U., Hutchings I.M. (2001). Investigation of surface treatments for galling protection of titanium alloys. Wear.

[5] Lu M., McCormick P., Zhao Y., Fan Z., Huang H. (2018). Laser deposition of compositionally graded titanium oxide on Ti6Al4V alloy. Ceram. Int..

[6] Slota J., Jurčišin M., Spišák E., Tomaáš M., Siser M. (2015). Experimental FLC determination of high strength steel sheet metal. Acta Metall. Slovaca.

[7] Slota J., Jurcisin M., Spisak E. (2012). Experimental and numerical analysis of local mechanical properties of drawn part. Key Eng. Mater..

[8] Makhkamov A., Wagre D., Baptista A.M., Santos A.D., Malheiro L. (2017). Tribology testing to friction determination in sheet metal forming processes. Ciência Tecnol. Mater..

[9] Trzepiecinski T. (2019). A study of the coefficient of friction in steel sheets forming. Metals.

[10] Zhou L., Gao K., Zheng X., Wang W., Wie X., Hua M. (2018). Developing of galling during the forming and its improvement by physical vapour depositing. Surf. Eng..

[11] Trzepieciński T., Fejkiel R. (2017). On the influence of deformation of deep drawing quality steel sheet on surface topography and friction. Tribol. Int..

[12] Trzepieciński T., Lemu H.G. (2020). Recent developments and trends in the friction testing for conventional sheet metal forming and incremental sheet forming. Metals.

[13] Shisode M., Hazrati J., Mishra T., de Rooij M., ten Horn C., van Breeck J., van den Boogard T. (2021). Modeling boundary friction of coated sheets in sheet metal forming. Tribol. Int..

[14] Ma J., Li H., Wang D., Fu W., Tao Z.J. (2018). Tribological behaviors in titanium sheet and tube forming at elevated temperatures: Evaluation and modeling. Int. J. Adv. Manuf. Technol..

[15] Ma J., Yang H., Li H., Wang D., Li G.J. (2015). Tribological behaviors between commercial pure titanium sheet and tools in warm forming. Trans. Nonferrous Met. Soc. China.

[16] Adamus J., Lackner J.M., Major Ł. (2013). A study of the impact of anti-adhesive coatings on the sheet-titanium forming processes. Arch. Civ. Mech. Eng..

[17] Więckowski W., Adamus J. (2013). Friction and wear testing of titanium and aluminium alloys. Obrobka Plast. Met..

[18] Jozwik J. (2018). Evaluation of tribological properties and condition of Ti6Al4V titanium alloy surface. Tech. Gaz..

[19] EN ISO 25178-2 (2012). Geometrical Product Specifications (GPS)—Surface Texture: Areal—Part 2: Terms, Definitions and Surface Texture Parameters.

[20] SAE J300 (2015). Engine Oil Viscosity Classification.

[21] SAE J306 (2017). Automotive Gear Lubricant Viscosity Classification.

[22] Bowden E.P., Tabor D. (1950). The Friction and Lubrication of Solids.

[23] Aksu G., Güzeller C.O., Eser M.T. (2019). The Effect of the Normalization Method Used in Different Sample Sizes on the Success of Artificial Neural Network Model. Int. J. Assess. Tools Educ..

[24] Burdack J., Horst F., Giesselbach S., Hassan I., Daffner S., Schöllhorn W.I. (2020). Systematic Comparison of the Influence of Different Data Preprocessing Methods on the Performance of Gait Classifications Using Machine Learning. Front. Bioeng. Biotechnol..

[25] Haykin S. (2009). Neural Networks and Learning Machines.

[26] Hertz J.A., Krogh A.S., Palmer R.G. (1991). Introduction to the Theory of Neural Computation.

[27] Trzepieciński T., Lemu H.G. (2012). Application of genetic algorithms to optimize neural networks for selected tribological tests. J. Mech. Eng. Autom..

[28] Cillaurren J., Galdos L., Sanchez M., Zabala A., de Argandoña E.S., Mendiguren J. Contact pressure and sliding velocity ranges in sheet metal forming simulations. Proceedings of the 24th International Conference on Material Forming ESAFORM 2021.

[29] Kirkhorn L., Frogner K., Andersson M., Stahl J.E. (2012). Improved tribotesting for sheet metal forming. Procedia CIRP.

[30] Hutchins I. (1992). Tribology: Friction and Wear of Engineering Materials.

[31] Hol J., Meinders V.T., Geijsealaers H.J.M., van den Boogaard A.H. (2015). Multi-scale friction modeling for sheet metal forming: The mixed lubrication regime. Tribol. Int..

[32] Shisoide M.P., Hazrati J., Mishra T., de Rooij M., van den Boogaard T. (2020). Modeling lubrication friction for sheet metal forming applications. Procedia Manuf..

[33] Budinski K.G. (1991). Tribological properties of titanium alloys. Wear.

[34] Kaivosoja E., Tianen V.M., Takakubo Y., Rajchel B., Sobiecki J., Kottinen Y.T., Takagi M., Affatato S. (2012). Materials used for hip and knee implants. Wear of Orthopaedic Implants and Artificial Joints.

[35] Andreasen J.L., Bay N., De Chiliffe L. (1998). Quantification of galling in sheet metal forming by surface topography characterization. Int. J. Mach. Tools Manuf..

[36] Dohda K., Yamamoto M., Hu C., Dubar L., Ehmann K.F. (2021). Galling phenomena in metal forming. Friction.

[37] Wojciechowski Ł. (2015). Problems of technological treatment of the surface layer in the aspect of the scuffing performance. Part I: The scuffing prognostication. Tribologia.

[38] Gåård A. (2018). Wear in Sheet Metal Forming. Licentiate Thesis.

[39] Vollertsen F. (2011). Size effects in micro forming. Key Eng. Mater..

[40] Podgornik B., Leskovšek V. (2015). Wear mechanisms and surface engineering of forming tools. Mater. Technol..

[41] Zhou Y., Jiang W., Chen W., Ji X.L., Jin Y.X., Wang S.Q. (2018). Modification of tribolayers of a titanium alloy sliding against a steel. J. Tribol..

[42] Bayer R.G. (2004). Mechanical Wear Fundamentals and Testing.

[43] Dong H., Dong H. (2010). Tribological properties of titanium-based alloys. Surface Engineering of Light Alloys.

[44] Rabinowicz E. (1954). Friction properties of titanium and its alloys. Met. Prog..

